# Effects of Electron Beam Irradiation on the Storage Stability and Quality Characteristics of Chicken and Duck Meat

**DOI:** 10.3390/foods14223867

**Published:** 2025-11-12

**Authors:** Kyu-Min Kang, Hack-Youn Kim

**Affiliations:** 1 Department of Animal Resource Science, Kongju National University, Yesan 32439, Republic of Korea; rbals15@naver.com; 2 Resource Science Research Institute, Kongiu National University, Yesan 32439, Republic of Korea

**Keywords:** irradiation dose, poultry meat, lipid oxidation, microbial safety, quality properties

## Abstract

This study evaluated the effects of low-dose electron beam irradiation (0, 1, 2, and 3 kGy) on storage stability and quality properties of chicken and duck breast meat. Five foodborne pathogens (*Salmonella typhimurium*, *Listeria monocytogenes*, *Staphylococcus aureus*, *Bacillus cereus*, and *Escherichia coli*) were inoculated into the samples and subjected to irradiation under vacuum packaging. The irradiated samples were vacuum-packed and stored at 4 °C. Microbial recovery, lipid and protein oxidation, physicochemical characteristics, and meat color were analyzed over 0, 1, and 2 weeks. A completely randomized design was used with five biological replicates (*n* = 5) per treatment, and each measurement was performed in triplicate (technical replicates). Electron beam treatment effectively reduced microbial counts, achieving complete inactivation of all pathogens except *Bacillus cereus* at 3 kGy. Irradiation resulted in significant reductions in pH and water-holding capacity (*p* < 0.05) while increasing thiobarbituric acid-reactive substances (TBARS) and volatile basic nitrogen (VBN) values, particularly in duck and chicken, respectively. Color parameters such as L* and b* decreased, while a*, chroma, and redness increased, with hue angle showing a decreasing trend. These changes were associated with myoglobin transformation and protein oxidation caused by irradiation-induced reactive oxygen species. Despite minor variations, proximate composition remained unaffected by irradiation. Overall, electron beam irradiation at doses up to 3 kGy effectively enhanced microbial safety without compromising nutritional quality, indicating its potential as a non-thermal preservation method for raw poultry meat products.

## 1. Introduction

Poultry meat is increasingly recognized as a healthy source of animal protein due to its high protein and low fat content, and its global consumption continues to rise steadily [1]. Beyond its functional health benefits, poultry accounts for the largest share of global meat production, owing to factors such as affordable pricing, ease of cooking and processing, and minimal religious restrictions [2]. Among poultry types, chicken and duck meat are particularly valued for their abundance of umami-enhancing amino acids, such as oligopeptides, and their high levels of unsaturated fatty acids, which are beneficial for cardiovascular health [3,4]. However, due to their high moisture and protein content, poultry products offer a favorable environment for microbial proliferation and are often regarded as natural growth media for microorganisms [5]. As such, proper caution must be taken to prevent foodborne illness through ingestion or handling [6]. Moreover, with the growing number of households raising companion animals such as dogs and cats, there has been a rise in the popularity of raw meat-based diets (RMBDs), further elevating public concern regarding microbial contamination [7].

In poultry meat, there is a potential risk of contamination by foodborne pathogens such as *Salmonella*, *Listeria monocytogenes*, and *Escherichia coli*, with cross-contamination from external sources after slaughter being a common route of infection [8]. The proliferation of such harmful microorganisms not only poses a threat to food safety by causing foodborne illnesses, but also contributes to the production of undesirable metabolic by-products during growth, leading to quality deterioration through lipid oxidation and protein degradation [9]. Traditionally, thermal processing has been the primary method used to ensure microbial safety [10]. While effective at inactivating pathogens, it often results in the degradation of heat-sensitive nutrients and undesirable changes in sensory attributes [11]. As a result, there is increasing interest in non-thermal food processing technologies as alternative methods that can preserve nutritional and sensory quality while still achieving microbial control [12]. These non-thermal methods aim to inactivate pathogens without the application of heat. Physical methods include high-pressure processing (HPP), ultraviolet (UV) radiation, and pulsed electric fields (PEF), whereas chemical approaches involve technologies such as non-thermal plasma and ozone treatment [13].

Among physical non-thermal processing technologies, electron beam irradiation (EBI) has gained considerable attention as a promising method for improving food hygiene and quality by inactivating microorganisms using high-energy electrons [14]. Unlike gamma irradiation, EBI does not rely on radioactive isotopes but instead accelerates electrons to near-light speeds to generate ionizing radiation, thereby minimizing the adverse effects on food quality and enhancing both scientific and industrial relevance [15]. Due to these advantages, EBI has been widely investigated for application in various food products, including meat, vegetables, fruits, and pet food, across a broad dose range—typically from 1 kGy up to over 20 kGy [16,17,18]. For instance, Wahyono et al. [19] conducted a meta-analysis on electron-beam irradiation in pork, applying doses ranging from 0 to 20 kGy. They reported improved microbiological safety by reducing populations of *Salmonella*, *E. coli* and *L. monocytogenes*, as well as enhanced oxidative stability in terms of TBARS and POV values. Similarly, Kwon et al. [20] evaluated the effects of 5 kGy electron-beam irradiation on ground chicken, pork, and beef. The study found increased lipid oxidation (particularly in chicken), and changes in volatile compound profiles, indicating that irradiation may affect sensory quality depending on species and processing conditions.

**Figure 1:**
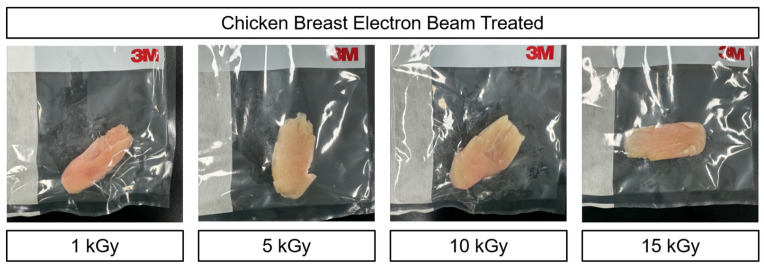
Color changes in chicken meat under various doses of electron beam irradiation: pre-test.

Nevertheless, despite its proven efficacy, continued research is needed to address consumer concerns and regulatory challenges regarding its safety and potential side effects [21]. Moreover, as shown in Figure 1, preliminary tests in this study revealed that higher irradiation doses noticeably altered the visual appearance of the meat, highlighting the need for focused analysis at lower doses to assess subtle quality changes. To further understand species-specific responses, chicken (light muscle) and duck (dark muscle) breast meats were selected due to their distinct myoglobin content and muscle fiber composition. Accordingly, this study focuses on assessing the effects of low-dose electron beam irradiation (0, 1, 2, and 3 kGy) on the storage stability and overall quality characteristics of chicken and duck meat, under the assumption that lower doses may induce fewer alterations in physicochemical properties while still ensuring microbial safety.

## 2. Materials and Methods

### 2.1. Preparation of Chicken and Duck Breast Meat

Chicken and duck breast samples were sourced 24 h post-slaughter from a commercial poultry processing company (Harim Co., Ltd., Iksan, Republic of Korea). All samples were transported under refrigerated conditions and processed immediately upon arrival. The outer surfaces of the samples were sanitized using 70% methyl alcohol, and the external layers were trimmed off. The samples were then divided into two groups: one set was inoculated with microorganisms for microbial recovery analysis, while the other set remained non-inoculated for physicochemical quality assessment. All samples underwent electron beam irradiation treatment. Following irradiation, the samples were stored at 4 °C under refrigerated conditions. Quality attributes were evaluated during storage, while microbial stability and shelf-life were analyzed at intervals of 0, 1, and 2 weeks.

### 2.2. Preparation of Bacterial Strains and Inoculum Suspension

In this study, the following bacterial strains were used: *Salmonella typhimurium* (*S. typhimurium*; ATCC 43971, ATCC 19525, ATCC DT104), *Listeria monocytogenes* (*L. monocytogenes*; ATCC 15313, ATCC 19111, ATCC 19115), *Staphylococcus aureus* (*S. aureus*; ATCC 13565 [SEA], ATCC 14458 [SEB], ATCC 19095 [SEC], ATCC 23235 [SED], FRI 913 [SEE]), *Bacillus cereus* (*B. cereus*; ATCC 14579 [diarrheal type], ATCC 10876 [emetic type]), and *Escherichia coli* (*E. coli*; ATCC 13715, ATCC 35150, ATCC 43889, ATCC 43890).

The preparation of bacterial strains and inoculum suspension was conducted according to the protocol of Won and Park [22], with slight modifications. Each strain was individually inoculated into 5 mL of tryptic soy broth (TSB; Difco, Chicago, IL, USA) and incubated at 37 °C for 24 h in an incubator (JSR, Gongju, Republic of Korea). After incubation, the cultures were transferred into sterile 50 mL conical tubes and centrifuged at 4000× *g* for 15 min at 4 °C using a centrifuge (Supra R22; Hanil Science, Gimpo, Republic of Korea) to harvest the bacterial cells in pellet form.

The resulting cell pellets were washed three times with sterile buffered peptone water (BPW; Difco, Detroit, MI, USA) to remove residual media components. The final inoculum was prepared by suspending the washed cells in 9 mL of BPW, adjusting the concentration to achieve approximately 10^8^–10^9^ CFU/mL for use in sample inoculation.

### 2.3. Bacterial Inoculation

A total of 0.1 mL of the prepared bacterial inoculum was aseptically applied onto chicken and duck breast meat samples (25 g, cut to dimensions of 5 × 10 cm) using a micropipette. The inoculum was distributed evenly over 15–20 spots across the sample surface. Following inoculation, the samples were air-dried under sterile conditions in a clean bench at 20 °C and then vacuum-packaged prior to electron beam irradiation.

### 2.4. Electron Beam Irradiation

Electron beam treatment was performed using a linear accelerator (EB-21-HEA10-01, EBtech, Daejeon, Republic of Korea) under controlled conditions (energy: 10 MeV; beam current: 436 mA; conveyor speed: 4.7 m/min). Samples were irradiated at 0, 1, 2, and 3 kGy doses for 10 min while maintained at 4 °C.

### 2.5. Microbial Recovery Analysis

For microbial recovery analysis, samples stored for 0, 1, and 2 weeks were homogenized with 0.1% buffered peptone water (BPW; Difco, Detroit, MI, USA) in sterile sample bags (1930F, 3M, Forest City, IA, USA) at ratios of 1:10 (sample:BPW). Homogenization was carried out for 1 min using a stomacher (WH4000-2751, 3M Korea, Seoul, Republic of Korea). Serial dilutions were prepared by adding 1 mL of homogenate to 9 mL of BPW and diluting as required.

Selective media were used for microbial enumeration as follows: *S. typhimurium* using MC-Media Pads (JNC Corp., Tokyo, Japan); *L. monocytogenes* with 3M™ Petrifilm™ (3M, Saint Paul, MN, USA); *S. aureus* using Microfast™ Staphylococcus aureus Count Plates (PKI-LR1005, PerkinElmer, Waltham, MA, USA); *E. coli* with Microfast™ Coliform & *E. coli* Count Plates (PKI-LR1007, PerkinElmer); and *B. cereus* using Microfast™ Bacillus Cereus Count Plates (PKI-LR1010, PerkinElmer).

One milliliter of each diluted sample was plated onto the respective media. *S. typhimurium*, *L. monocytogenes*, *E. coli* and *S. aureus* were incubated at 37 °C for 24 h, while *B. cereus* were incubated at 30 °C for 24 h. Colony counts were expressed as logarithmic colony-forming units per gram (log CFU/g).

### 2.6. Thiobarbituric Acid-Reactive Substances (TBARS) Analysis

TBARS value was determined following the method described by Kang et al. [23], with slight modifications. Briefly, 5 g of sample was homogenized with 12.5 mL of 10% perchloric acid (PCA) solution and 200 µL of 0.3% butylated hydroxytoluene (BHT) using a homogenizer (AM-5, Nihonseiki, Tokyo, Japan) for 1 min. The homogenate was then filtered through Whatman No. 1 filter paper (GE Healthcare, Chicago, IL, USA). A 5 mL aliquot of the filtrate was mixed with 5 mL of 0.02 M thiobarbituric acid (TBA) solution and incubated in a water bath at 100 °C (JSWB-30T, JSR, Gongju, Republic of Korea) for 10 min. After the reaction, absorbance was measured at 532 nm using a multi-mode microplate reader (SpectraMax iD3, Molecular Devices, San Jose, CA, USA). The amount of malondialdehyde (MDA) formed was calculated from a standard curve prepared using 1,1,3,3-tetraethoxypropane. TBARS values were expressed as mg MDA per kg of sample.

### 2.7. Volatile Basic Nitrogen (VBN) Analysis

VBN content was determined according to the method of Lee and Kim [24], with slight modifications. Ten grams of sample was weighed and homogenized with 30 mL of distilled water at 10,923× *g* for 1 min. The homogenate was then diluted with distilled water to a final volume of 100 mL and filtered through Whatman No. 1 filter paper (GE Healthcare). For the Conway micro-diffusion method, 1 mL of the filtrate was added to the outer chamber of a Conway unit, and 1 mL of 0.01 N boric acid (H_3_BO_3_) was added to the inner chamber, followed by the addition of 100 µL of Conway reagent. Then, 1 mL of 50% potassium carbonate (K_2_CO_3_) was added to the outer chamber. The unit was immediately sealed and incubated at 37 °C for 2 h. After incubation, the contents of the inner chamber were titrated with 0.02 N sulfuric acid (H_2_SO_4_). The VBN value (mg/100 g) was calculated using the following equation:

$$\mathrm{VBN}\;\left(\mathrm{mg}/100\;g\right)=\frac{(A-B)\;\times \;(f\;\times \;N\;\times \;14.007\;\times \;100\;\times \;c)}{S}$$

where

A = volume of H_2_SO_4_ used for sample titration (mL)

B = volume of H_2_SO_4_ used for blank titration (mL)

f = titrant factor

N = normality of H_2_SO_4_ (0.02 N)

c = dilution factor

S = sample weight (g)

### 2.8. Proximate Composition Analysis

The proximate composition of the samples was determined according to the AOAC methods [25]. Moisture content was measured using the atmospheric drying method at 105 °C (AOAC 950.46), crude protein content was analyzed by the Kjeldahl method (AOAC 992.15), crude fat content was determined using the Soxhlet extraction method (AOAC 960.39), and crude ash content was measured by dry ashing method at 550 °C (AOAC 920.153). All results were expressed as percentages (%).

### 2.9. pH

A 4 g sample was homogenized with 16 mL of distilled water using an Ultra Turrax (6991× *g*, 1 min), and pH was determined with a glass electrode pH meter (S220, Mettler-Toledo, Greifensee, Switzerland)

### 2.10. Water Holding Capacity (WHC) Analysis

WHC was assessed using the filter paper press method [23]. A 0.3 g of the sample was placed on Whatman No. 1 filter paper (GE Healthcare) and pressed for 3 min. The WHC was calculated with the following formula:

$$\mathrm{WHC}\;\left(\%\right)\;=\;\frac{\;\mathrm{Meat}\;\mathrm{area}\;({\mathrm{mm}}^{2})}{\mathrm{Exuded}\;\mathrm{water}\;\mathrm{area}\;({\mathrm{mm}}^{2})}\;\times \;100$$

### 2.11. Shear Force Analysis

Samples (1 × 1 × 2 cm^3^) were tested using a texture analyzer (TA 1, Lloyd, Largo, FL, USA) equipped with a V-blade. The test was conducted at 21.0 mm/s speed, 22.0 mm distance, and 5.6 N. Results were reported in kgf.

### 2.12. Color Analysis

Color was measured using a colorimeter (CR-10, Minolta, Tokyo, Japan) with an 8 mm aperture, D65 illuminant, 2° observer, and pulsed xenon lamp. After calibration with a standard white plate (L* + 97.83, a* − 0.43, b* + 1.98), values for L*, a*, and b* were recorded. Hue angle and chroma values were calculated using the following formula:

$$\mathrm{Hue}\;\mathrm{angle}=\tan^{-1}b^{*}/a^{*},\;\mathrm{Chroma}={\left(a^{*2}+b^{*2}\right)}^{\frac{1}{2}}$$

### 2.13. Statistical Analysis

All experimental treatments were conducted using five biological replicates (*n* = 5), where each replicate consisted of independently prepared and irradiated meat samples. For each biological replicate, all analytical measurements—including microbial recovery, TBARS, VBN, proximate composition, pH, WHC, shear force, and color—were conducted in triplicate as technical replicates (*n* = 3), and the average of the technical replicates was used for statistical analysis. Statistical analyses were verified using one-way analysis of variance (ANOVA) and Tukey’s multiple comparisons test in the GraphPad Prism 10 for windows (version 10.4.0; GraphPad Prism Software Inc., San Diego, CA, USA) at a significant level of *p*-value < 0.05.

## 3. Results and Discussion

### 3.1. Microbial Recovery

**Figure 2:**
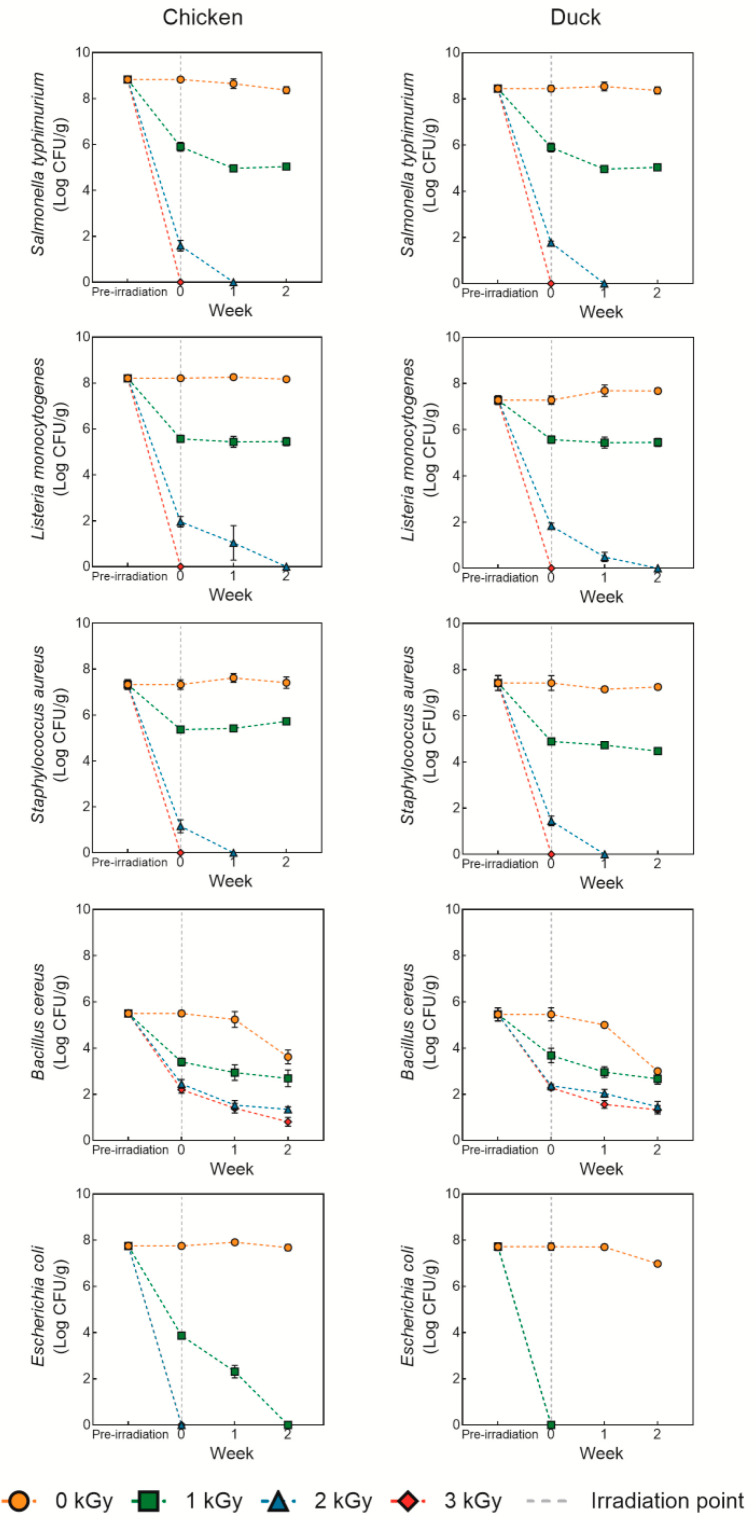
Microbial recovery of chicken and duck meat under various doses of electron beam irradiation.

Figure 2 shows the microbial recovery of chicken and duck meat under various doses of electron beam irradiation. Overall, the survival rates of *Salmonella typhimurium*, *Listeria monocytogenes*, *Staphylococcus aureus*, and *Escherichia coli* decreased as the irradiation dose increased, with complete inactivation observed at doses of 3 kGy or higher (*p* < 0.05). Similar results were reported by Lung et al. [26], who found that electron beam irradiation effectively inhibited or eliminated most microorganisms in meat, dairy, and seafood even at low doses. Electron beam irradiation causes double-strand breaks in microbial DNA via high-energy electrons, inhibiting replication and survival. Additionally, it generates reactive oxygen species (ROS), which induce oxidative stress, leading to damage in cellular structures and metabolic functions [27]. Although Gram-negative bacteria (*Salmonella*, *E. coli*) and Gram-positive bacteria (*Listeria*, *Staphylococcus*) differ in cell wall thickness, both are relatively susceptible to DNA damage and ROS-induced stress caused by electron beams, making them vulnerable even at lower doses [28]. Once DNA damage and oxidative stress impair the bacterial repair mechanisms, these pathogens fail to survive under refrigerated conditions (below 10 °C), leading to their gradual inactivation [29]. In contrast, *Bacillus cereus* exhibited a decreasing trend in survival with increasing irradiation dose but were not completely inactivated even at 3 kGy. This resistance is attributed to the structural characteristics of spore-forming bacteria. *Bacillus* spores consist of multiple protective layers, including the cortex, outer membrane, coats, and exosporium, which contribute to their resistance against radiation and similar treatments [30]. Additionally, small acid-soluble spore proteins (SASP) within the spores bind tightly to DNA, enhancing their resistance, and later serve as energy sources during germination, facilitating the outgrowth of vegetative cells [31]. Therefore, for effective control of *Bacillus* spp., combined sterilization technologies may be necessary [32]. In conclusion, electron beam irradiation at doses of 3 kGy or higher is effective in inactivating most foodborne pathogens, but additional intervention strategies are required to suppress spore-forming bacteria such as *Bacillus*. Also, from an industrial perspective, electron beam irradiation offers a promising hygienic processing strategy, particularly for cold-chain meat distribution. However, its broader application will depend on cost-effectiveness, regulatory approval, and alignment with existing processing systems.

### 3.2. TBARS and VBN

**Figure 3:**
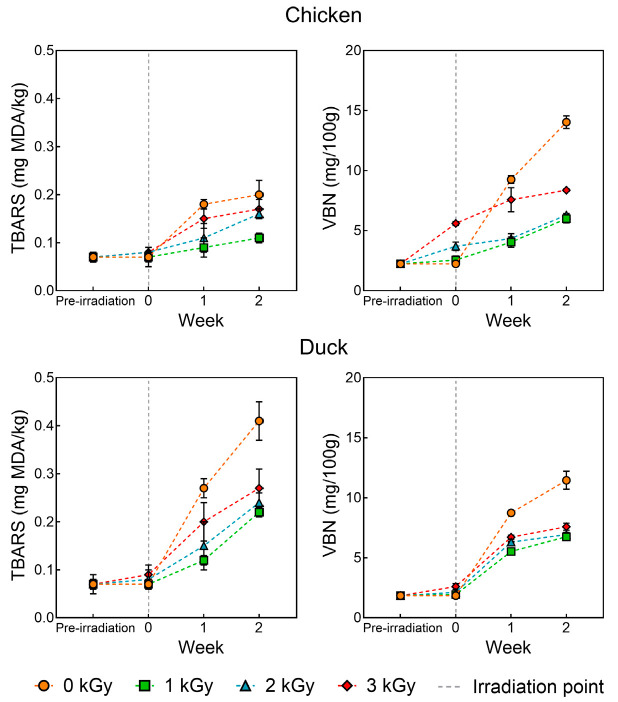
TBARS and VBN of chicken and duck meat under various doses of electron beam irradiation.

Figure 3 presents the TBARS and VBN of chicken and duck meat under various doses of electron beam irradiation. In general, as the storage period progressed, both lipid oxidation and protein degradation increased, with the control group showing the most significant and rapid changes (*p* < 0.05). Among the irradiated groups, higher doses of electron beam irradiation led to greater increases in both indices. Lipid oxidation was more pronounced in duck meat samples, whereas protein degradation progressed more rapidly in chicken meat. Similar findings were reported by Arshad et al. [33], who observed that both TBARS and VBN values in gamma-irradiated chicken increased with irradiation dose and storage time. In meat, lipid oxidation is primarily influenced by fat content and fatty acid composition, while protein degradation is largely driven by microbial activity and endogenous proteolytic enzymes [19]. The current results also align with the proximate composition analysis, indicating that the differences in moisture, fat, and protein contents between chicken and duck meat contributed to the differences in lipid and protein degradation rates. Electron beam irradiation generates high-energy electrons that ionize water molecules in the sample, leading to the production of ROS [34]. Among these, free radicals initiate lipid oxidation by abstracting hydrogen atoms from methylene groups of polyunsaturated fatty acids, forming conjugated dienes. These dienes subsequently react with oxygen to form highly reactive lipid radicals, which then extract hydrogen atoms from adjacent fatty acid chains, propagating a chain reaction [35]. This process accelerates lipid peroxidation in meat products. Moreover, electron beams also induce protein structural modifications, leading to an increase in VBN values. Notably, this increase is not primarily due to microbial activity but rather due to protein degradation caused by irradiation [36]. Free radicals generated by electron beams can disrupt disulfide bonds and other cross-links involved in maintaining secondary and tertiary protein structures, resulting in protein denaturation [37]. In conclusion, while electron beam treatment was effective in suppressing microbial growth and thus delayed microbial spoilage, the formation of ROS and free radicals during irradiation promoted lipid and protein oxidation. Therefore, it is necessary to consider combined treatments that can effectively mitigate oxidative damage induced during electron beam processing.

### 3.3. Proximate Composition

Table 1 shows the proximate composition of chicken and duck meat under various doses of electron beam irradiation. Moisture, fat, and ash contents were significantly higher in duck samples (*p* < 0.05), while protein content was significantly higher in chicken samples (*p* < 0.05). No significant differences were observed according to irradiation dose, indicating that electron beam treatment did not notably affect the proximate composition of the meat. This finding aligns with previous studies reporting that electron beam irradiation does not significantly alter the basic composition or sensory attributes of food products [38,39]. These results suggest that while lipid oxidation and protein denaturation may occur due to irradiation, the actual amounts of fat and protein remain unchanged. Therefore, the current findings imply that electron beam irradiation can be utilized for microbial control in both chicken and duck meat without compromising their nutritional value, supporting its potential as a safe and effective non-thermal food processing technology.

### 3.4. pH, WHC, and Shear Force

Table 2 shows the pH, WHC, and shear force of chicken and duck meat under various doses of electron beam irradiation. The pH value was significantly higher in chicken samples compared to duck samples (*p* < 0.05), and it showed a gradual decreasing trend as the irradiation dose increased. Similarly, Ham et al. [40] reported a significant reduction in pH after applying electron beam irradiation at doses of 2–6 kGy to pork sausages. This decrease in pH may be attributed to the radiolysis of water molecules during irradiation, which generates hydrogen ions (H^+^). These H^+^ ions are not immediately neutralized and tend to remain in the sample, resulting in a lower pH [41]. Furthermore, the recombination rate of H^+^ and OH^−^ ions generated by irradiation is slower than that under equilibrium conditions, leading to prolonged retention of H^+^ and contributing to the observed pH reduction. On the other hand, studies by An et al. [42] and Arshad et al. [43] have reported either no change or even an increase in pH following low-dose electron beam treatment. These discrepancies suggest that factors such as irradiation dose, moisture content of the food, buffering capacity, and the timing of pH measurement may influence the outcome. Therefore, further investigation is needed to elucidate the underlying mechanisms of pH changes, particularly under low-dose electron beam irradiation.

WHC was significantly higher in duck meat samples compared to chicken meat samples (*p* < 0.05), and showed a significant decreasing trend with increasing irradiation dose (*p* < 0.05). A similar trend was reported by Orynbekov et al. [44], who found that WHC decreased in chicken meat as the electron beam dose increased. This reduction in WHC may be attributed to structural changes within the muscle tissue caused by irradiation, such as myofibrillar contraction, increased membrane permeability, and protein denaturation [45]. Moreover, changes in protein structure induced by electron beam treatment have been shown to increase the mobility between free and bound water, thereby reducing the meat’s water-holding ability [46]. Supporting this, low-field nuclear magnetic resonance (LF-NMR) analysis revealed a decrease in immobilized water and an increase in free water, indicating that water redistribution due to protein structural alterations may contribute to WHC deterioration [47]. Additionally, the higher WHC observed in duck meat compared to chicken may be attributed to differences in inherent fat content and muscle fiber structure [39]. These findings suggest that setting an optimal irradiation dose is essential for maintaining the quality of meat products, and further research is warranted to develop auxiliary technologies aimed at preserving WHC during electron beam treatment.

Shear force did not show any statistically significant differences based on species or irradiation dose. This is consistent with the findings of Gomes et al. [48], who also reported no significant effect of electron beam irradiation on the shear force of chicken meat. However, the overall shear force values exhibited a decreasing trend, which may be associated with structural changes caused by reactive oxygen species (ROS) generated during irradiation [49]. Specifically, ROS can induce oxidative modifications such as protein carbonylation by altering the secondary and tertiary structures of proteins, thereby weakening the bonding within myofibrillar proteins and ultimately resulting in a softer meat texture. Supporting this, Wang et al. [50] demonstrated that increased carbonyl content in irradiated meat proteins was closely related to reduced shear force. Although no statistically significant differences were observed, these findings suggest that electron beam irradiation may contribute to meat tenderization through protein oxidation and structural alterations. Therefore, further investigation into the relationship between oxidative changes and texture attributes is warranted to better understand the mechanisms underlying irradiation-induced tenderness. In parallel, practical application of this technology must also consider consumer acceptance, which remains a major challenge due to persistent misconceptions about irradiation. Educational efforts and transparent labeling will be essential to build public trust and support wider adoption.

### 3.5. Color

**Figure 4:**
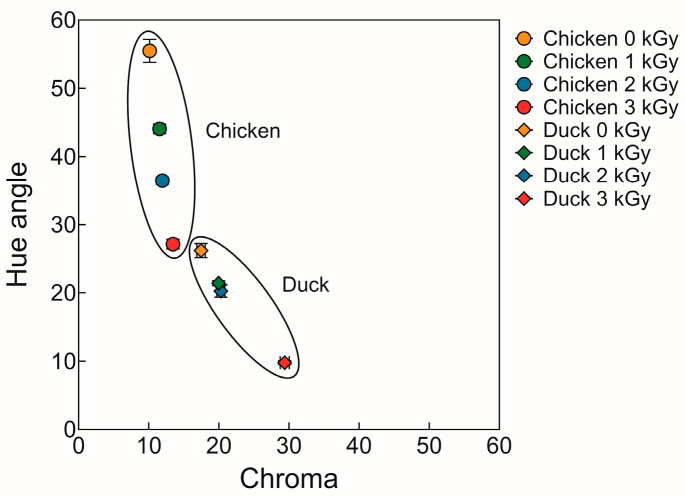
Hue angle and chroma of chicken and duck meat under various doses of electron beam irradiation.

Table 3 and Figure 4 show the color of chicken and duck meat under various doses of electron beam irradiation. Lightness (L*) and yellowness (b*) values were significantly higher in chicken samples compared to duck samples (*p* < 0.05), whereas redness (a*) was significantly greater in duck meat (*p* < 0.05). In addition, as the irradiation dose increased, both L* and b* values showed a decreasing trend, while a* values tended to increase. These findings are in agreement with the study by Lewis et al. [51], which reported similar changes in color attributes of chicken breast following electron beam irradiation—namely, a decrease in lightness and yellowness and an increase in redness with higher irradiation doses. These color changes can be attributed to the state of myoglobin in meat. Electron beam irradiation promotes the conversion of metmyoglobin to oxymyoglobin, thereby increasing the a* value [19]. Moreover, under vacuum conditions, electron beam treatment can lead to the formation of carbon monoxide (CO), which binds with myoglobin to form carboxymyoglobin—a pigment that visually resembles the bright red color of oxymyoglobin [52]. Since the samples in this study were vacuum-packaged prior to irradiation, it is likely that the enhancement in redness is predominantly due to the formation of carboxymyoglobin.

Moreover, with increasing irradiation doses, hue angle values showed a decreasing trend, while chroma values tended to increase. Notably, the hue angle and chroma values of the 3 kGy-irradiated chicken samples were similar to those of the non-irradiated duck samples. A decrease in hue angle indicates a shift toward a redder or reddish-brown coloration, whereas an increase in chroma reflects greater color saturation—suggesting that the meat color became more vivid and intense following irradiation [53]. These changes may be attributed to structural alterations in muscle proteins caused by electron beam treatment, which can influence water retention and the surface’s light-reflecting properties, thereby enhancing color intensity [54]. This observation implies that while duck meat inherently exhibits a darker, more reddish hue due to higher pigment content, chicken meat subjected to irradiation may undergo color changes that make it visually resemble duck meat. Therefore, irradiation dose may potentially act as a tool for modulating or harmonizing color differences between poultry species.

## 4. Conclusions

This study highlights the potential of low-dose electron beam irradiation (up to 3 kGy) as a non-thermal intervention to enhance the microbial safety of chicken and duck breast meat. While irradiation effectively reduced microbial loads without significantly altering proximate composition, species- and dose-dependent changes in physicochemical and quality attributes were observed. These findings suggest that irradiation may contribute to improved shelf life and meat texture; however, associated changes in pH, WHC, and color imply a need for careful dose optimization. Overall, electron beam treatment offers a promising strategy for poultry meat preservation, provided that quality trade-offs are managed through integrated processing approaches. Nonetheless, its future industrial applicability will rely on optimizing dose levels, improving economic feasibility, and fostering consumer acceptance through proper education and clear communication.

## Figures and Tables

**Table 1:** Proximate composition of chicken and duck meat under various doses of electron beam irradiation.

Trait (%)		Dose (kGy)			
		0 (Control)	1	2	3
Moisture	Chicken	75.11 ± 0.71 ^Y^	75.07 ± 0.71 ^Y^	74.93 ± 0.01 ^Y^	74.86 ± 0.01 ^Y^
	Duck	76.61 ± 0.35 ^X^	76.38 ± 0.10 ^X^	76.78 ± 0.24 ^X^	76.72 ± 0.03 ^X^
Protein	Chicken	22.13 ± 0.46 ^X^	22.20 ± 0.58 ^X^	22.18 ± 0.38 ^X^	22.20 ± 0.37 ^X^
	Duck	19.56 ± 0.68 ^Y^	19.51 ± 0.57 ^Y^	19.55 ± 0.86 ^Y^	19.48 ± 0.39 ^Y^
Fat	Chicken	0.40 ± 0.04 ^Y^	0.41 ± 0.01 ^Y^	0.41 ± 0.05 ^Y^	0.42 ± 0.07 ^Y^
	Duck	1.20 ± 0.06 ^X^	1.28 ± 0.17 ^X^	0.94 ± 0.19 ^X^	0.93 ± 0.18 ^X^
Ash	Chicken	1.22 ± 0.02 ^Y^	1.24 ± 0.03 ^Y^	1.24 ± 0.02 ^Y^	1.26 ± 0.01 ^Y^
	Duck	1.54 ± 0.07 ^X^	1.60 ± 0.08 ^X^	1.66 ± 0.17 ^X^	1.69 ± 0.08 ^X^

All values are means ± standard deviation (*n* = 5). ^X,Y^ Means in the same column with different letters are significantly different (*p* < 0.05).

**Table 2:** pH, WHC, and shear force of chicken and duck meat under various doses of electron beam irradiation.

Trait		Dose (kGy)			
		0 (Control)	1	2	3
pH	Chicken	5.98 ± 0.01 ^Xa^	5.98 ± 0.01 ^Xa^	5.96 ± 0.01 ^Xb^	5.96 ± 0.01 ^Xb^
	Duck	5.84 ± 0.03 ^Ya^	5.81 ± 0.03 ^Yab^	5.77 ± 0.02 ^Yb^	5.77 ± 0.02 ^Yb^
WHC (%)	Chicken	50.81 ± 0.48 ^Ya^	45.15 ± 0.33 ^b^	41.36 ± 0.71 ^Yc^	34.95 ± 0.71 ^Yd^
	Duck	55.71 ± 0.43 ^Xa^	46.12 ± 0.70 ^b^	42.82 ± 0.54 ^Xc^	40.18 ± 0.40 ^Xd^
Shear force (kgf)	Chicken	1.58 ± 0.18	1.47 ± 0.43	1.47 ± 0.08	1.27 ± 0.17
	Duck	1.64 ± 0.33	1.48 ± 0.39	1.47 ± 0.07	1.34 ± 0.08

All values are means ± standard deviation (*n* = 5). ^X,Y^ Means in the same column with different letters are significantly different (*p* < 0.05). ^a–d^ Means in the same row with different letters are significantly different (*p* < 0.05).

**Table 3:** Color of chicken and duck meat under various doses of electron beam irradiation.

Trait		Dose (kGy)			
		0 (Control)	1	2	3
CIE L^*^	Chicken	50.63 ± 0.51 ^X^	50.53 ± 0.25 ^X^	50.02 ± 0.72 ^X^	49.73 ± 0.49 ^X^
	Duck	40.67 ± 0.83 ^Ya^	39.70 ± 0.20 ^Yab^	39.63 ± 0.52 ^Yab^	38.47 ± 0.86 ^Yb^
CIE a^*^	Chicken	5.83 ± 0.36 ^Yd^	8.30 ± 0.30 ^Yc^	9.50 ± 0.29 ^Yb^	11.97 ± 0.45 ^Ya^
	Duck	15.67 ± 0.67 ^Xc^	18.60 ± 0.10 ^Xb^	19.03 ± 0.58 ^Xb^	28.97 ± 0.64 ^Xa^
CIE b^*^	Chicken	8.33 ± 0.05 ^Xa^	8.03 ± 0.12 ^Xb^	7.13 ± 0.17 ^c^	6.13 ± 0.13 ^Xd^
	Duck	7.70 ± 0.10 ^Ya^	7.30 ± 0.17 ^Yab^	7.03 ± 0.31 ^b^	5.00 ± 0.10 ^Yc^

All values are means ± standard deviation (*n* = 5). ^X,Y^ Means in the same column with different letters are significantly different (*p* < 0.05). ^a–d^ Means in the same row with different letters are significantly different (*p* < 0.05).

## Data Availability

The original contributions presented in this study are included in the article. Further inquiries can be directed to the corresponding author.
